# Functional Recognition by CD8+ T Cells of Epitopes with Amino Acid Variations Outside Known MHC Anchor or T Cell Receptor Recognition Residues

**DOI:** 10.3390/ijms21134700

**Published:** 2020-07-01

**Authors:** Kirsty L. Wilson, Sue D. Xiang, Magdalena Plebanski

**Affiliations:** 1 School of Health and Biomedical Sciences, RMIT, 3083 Bundoora, Australia; kirsty.wilson2@rmit.edu.au; 2 Department of Immunology and Pathology, Monash University, 3004 Melbourne, Australia; sue.xiang@monash.edu

**Keywords:** altered peptides, alanine screening, nanoparticles, delivery system, T cell, vaccine design, cross-reactivity

## Abstract

Peptide-based vaccines can be safer and more cost effective than whole organism vaccines. Previous studies have shown that inorganic polystyrene nanoparticles (PSNPs) covalently conjugated to the minimal immunodominant peptide epitope from murine liver stage malaria (SYIPSAEKI) induced potent CD8+ T cell responses. Many pathogens, including malaria, have polymorphic T cell epitope regions. Amino acid changes in positions that are contact residues for the T cell receptor (TCR) often alter the specific cross-reactivity induced by the peptide antigen, and it is largely assumed that changes outside of these residues have little impact. Herein, each amino acid residue (except major histocompatibility complex (MHC) anchors) was systematically changed to an alanine. Peptide epitopes with altered amino acids outside T cell contact residues were still recognized by T cells induced by PSNPs-SYIPSAEKI (KI) vaccines, albeit at lower levels, except for the variant SYIPSAAKI (A7). PSNPs-SYIPSAAKI vaccines further elicited high responses to the index KI peptide. None of the epitopes displayed altered peptide ligand (APL) antagonism in vitro, and re-stimulating SYIPSAEKI and SYIPSAAKI together synergistically enhanced IFN-γ production by the T cells. These results show epitope variation in non-TCR recognition residues can have effects on T cell reactivity, suggesting that such natural variation may also be driven by immune pressure. Additionally, when re-modelling peptides to enhance the cross-reactivity of vaccines, both TCR recognition and non-recognition residues should be considered.

## 1. Introduction

Attenuated live pathogen vaccines often activate potent immune responses, however, these vaccines carry the risk of pathogen reactivation and cannot be given to immunocompromised or vulnerable populations. Subunit vaccines, including peptide-based vaccines, can be a safer option as they contain only the immune activating antigen and well-defined adjuvant/carrier components. The main advantage of peptide vaccines lies in their high specificity and the ability to induce potent T cells responses, particularly CD8+ T cells, which is not easy with other types of vaccines. Peptide based vaccines are also stable and generally have a simple structure and are easier to manufacture, making them cheaper alternatives. There is also the option of including single or multiple epitopes into the vaccine preparation, depending on the immune response required.

One disadvantage of peptide-based vaccines is that they are less immunogenic and need delivery strategies or adjuvant systems to activate strong T cell responses. Current licensed adjuvants or vaccine delivery vehicles capable of inducing such T cell responses include MF59 [1] and other oil or squalene-based emulsion adjuvants (i.e., adjuvant system AS02) [2], as well as viral vectored vaccines (i.e., Chimpanzee adenoviral (ChAd) and modified vaccinia Ankara (MVA)) [3]. Delivery systems such as virus like particles (VLPs) and immune stimulating complexes (ISCOMs) are also potent inducers of T cell responses. One natural delivery system is through the use of ex vivo peptide pulsed dendritic cells (DCs), which is mostly used in immune-therapies to boost recognition and activation of tumor antigens in cancer patients [4]. Autologous DCs naturally induce T cell responses, without the need for an additional adjuvant, though as it is a highly individualized therapy, it is expensive and would not be suitable for widespread use in developing countries. 

A similarly potent experimental adjuvant is Montanide ISA 720, a water in oil emulsion [5]. This emulsion has been used in clinical trials with peptides from liver stage malaria antigens [6,7]. A similar preparation, Montanide ISA 51 has been studied for its use as an adjuvant for delivery of tumor peptide antigens, due to its capability to induce CD8+ T cells [8,9]. Montanide is a strong adjuvant in comparison to others, though it has been known to cause local reactions at the injection site [10]. Another experimental vaccine delivery system is the use of nanoparticles to induce immune responses. Peptides can be simply mixed with the nanoparticle, incorporated into the nanoparticle, presented on the surface of the nanoparticle or covalently bound to the surface of the nanoparticle. Utilizing nanoparticles to induce potent immune responses is a growing field, especially for peptide-based vaccines [11,12]. Inorganic polystyrene nanoparticles (PSNPs) in the viral size range (40–50 nm) are inert and induce both antibodies and CD8+ T cells to peptide antigens [13,14,15,16]. 

Subunit vaccines that induce CD8+ T cells are highly regarded for diseases such as HIV, malaria, and cancers where cytotoxic T lymphocytes (CTLs) are required to kill infected cells. Much of the research is being done on what makes a highly specific and immunogenic epitope for vaccines, including the possibility of designing or engineering epitopes. T cells recognize peptide epitopes presented by major histocompatibility complex (MHC) molecules. For CD8+ T cell epitopes, 8-9mer peptides are presented within MHC class I molecules. Given that CD8+ T cell epitopes are so short, it is important to understand the relative impact of each amino acid position along the epitope on the resulting immune response. It is known that the MHC anchor residues for a 9mer epitope are positions 2 and 9, with the TCR contact sites within residues 3–8 [17]. By changing each amino acid along a peptide, the influence of each position can be elucidated. This can be achieved through a process of alanine screening, by changing each amino acid position along an epitope to an alanine, to assess the resulting effects on recognition and immunogenicity. Alanine is chosen as it is a small amino acid that contains non-reactive side chains, allowing deduction of the function of the substituted amino acid and its side chains.

Knowledge of the importance of amino acid changes along a peptide also comes from the use of altered peptide ligands (APLs). APLs are epitope modifications based on amino acid substitutions [18]. Single or multiple amino acid changes can influence the response to foreign peptides in a population of pathogens [18]. One feature of APLs is that they can aid in immune evasion of pathogens and can be influenced by immune selection pressure for immunodominant epitopes [19,20]. Malaria parasites are well known to contain APLs to escape the immune response. This can be modelled in animal models of malaria using the CD8+ T cell epitope from the *Plasmodium berghei* liver stage circumsporozoite protein, SYIPSAEKI (KI) [21,22].

Previously it has been shown that minimal immunodominant peptide epitopes from *Plasmodium berghei* (SYIPSAEKI) covalently conjugated to PSNPs induce functional IFN-γ T cell responses comparable to Montanide after two immunizations [23]. Within this H2-kd restricted peptide, the lysine (K) at position 8 was identified as being a key T cell recognition residue, and changing this residue to other amino acids, especially alanine, drastically reduced the T cell response [24]. However, less is known about the response to altered peptide residues outside the key T cell contact sites and if their modification would affect vaccine induced responses. 

Herein we assess the T cell response using model SYIPSAEKI epitopes that have been systematically altered by changing each position along the peptide, outside the T cell recognition and MHC anchor sites (the Y at position 2 and I at position 9) [24,25,26], to an alanine (SYIPSAE**A**I, SYIPSA**A**KI, SYIP**A**AEKI, SYI**A**SAEKI, SY**A**PSAEKI, **A**YIPSAEKI), in different vaccine delivery systems (Montanide, PSNPs and peptide pulsed dendritic cells (DCs)). Such research will help us to have a better understanding of the MHC-TCR recognition requirements and design better strategies for peptide-based vaccines to achieve a desired immune response.

## 2. Results

### 2.1. Peptide Antigen Delivery via Conjugation to Nanoparticles (PSNPs) Preserves the Moderate Cross-Reactivity to Alanine Altered Peptide Variants of SYIPSAEKI

Previously it has been observed that Montanide and conjugated PSNPs induce high magnitude CD8+ T cell responses to the minimal peptide immunodominant epitope from P. *berghei*, SYIPSAEKI [23]. Additionally, it was observed that position 8 along this peptide (lysine), was a key functional T cell contact residue, as changing this residue to other amino acids, particularly alanine (A), resulted in a complete loss of reactivity [24]. Therefore, we wanted to alanine screen the epitope by changing each of the other amino acids outside the T cell reactive site along the peptide to an alanine, apart from the MHC binding sites of tyrosine (Y) and isoleucine (I). This resulted in the following peptides; SYIPSAE**K**I (native epitope—KI), SYIPSAE**A**I (confirmed decrease in reactivity—A8), SYIPSA**A**KI (A7), SYIP**A**AEKI(A5), SYI**A**SAEKI (A4), SY**A**PSAEKI (A3), **A**YIPSAEKI (A1). 

**Figure 1:**
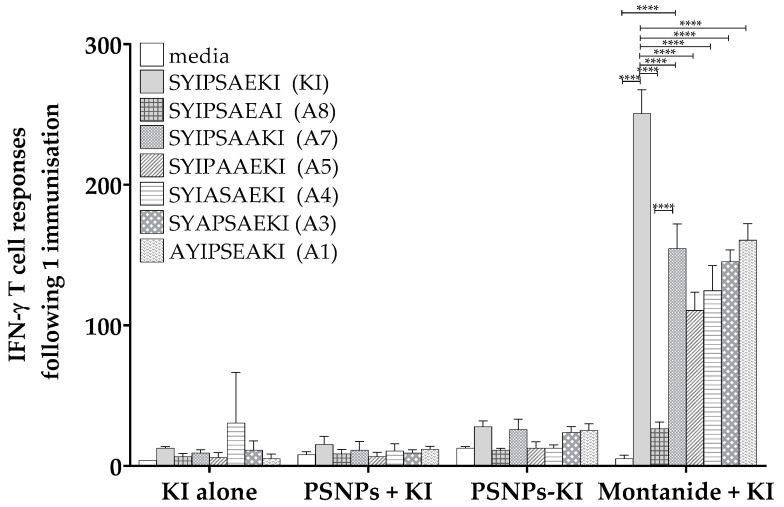
Moderate cross-reactivity to restimulated alanine altered variants of SYIPSAEKI. The 6–8 week old BALB/c mice were immunized once with KI peptide alone (25 µg KI per mouse), PSNPs plus KI or KI conjugated to PSNPs (PSNPs + KI, PSNPs-KI, respectively, 25 µg per mouse KI and 1% solids PSNPs for both groups), or Montanide plus KI (25 µg KI per mouse in 70%/30% *v*/*v* Montanide/Phosphate buffered saline (PBS)). Two weeks after the last immunization, splenocytes from immunized mice were harvested and restimulated with altered peptides in an in vitro ELISpot assay for IFN-γ. Data shown as mean +/- SD of spot forming units (SFU)/million cells per assay triplicates (pooled cells from 3–4 mice per group). * *p* < 0.05, ** *p* < 0.01, *** *p* < 0.001, **** *p* < 0.0001.

Mice were immunized once with the native epitope SYIPSAEKI (herein termed KI) adjuvanted with Montanide, or conjugated to PSNPs, and T cell responses were examined via IFN-γ ELISpot assay. Cells from immunized mice were restimulated with each of the alanine screened peptides to examine reactivity. Following one immunization, KI peptide alone or PSNPs plus KI did not induce strong T cell responses, and immunizing with PSNPs conjugated to KI only elicited small responses to KI. After one immunization with Montanide plus KI, as expected, there were high magnitude responses to KI itself, significantly higher than to all other epitopes (Figure 1). In vitro restimulation with the additional altered peptides, A7, A5, A4, A3, A1, significantly decreased reactivity compared to KI (Figure 1). All variants apart from A8 significantly restimulated cross-reactive responses compared to background, in the Montanide plus KI immunized group (Figure 1). 

**Figure 2:**
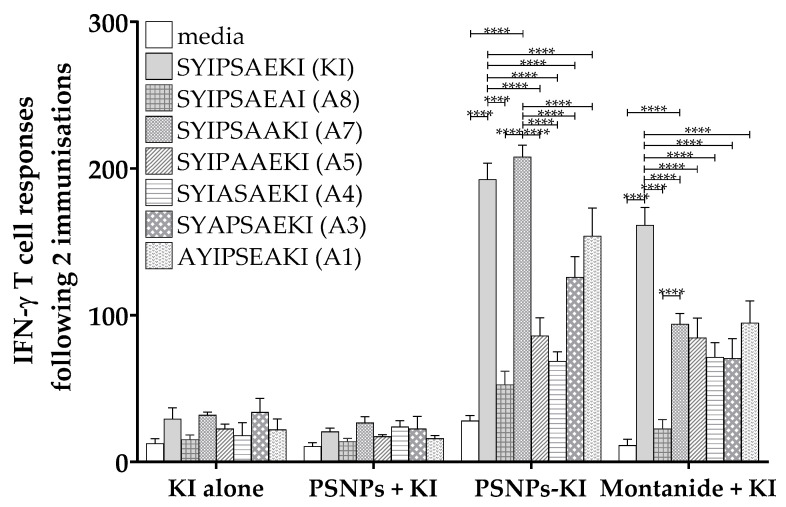
Different patterns of IFN-γ reactivity to alanine altered peptide variants with Montanide adjuvanted or conjugated PSNPs immunized mice. The 6-8 week old BALB/c mice were immunized twice with KI peptide alone (25 µg KI per mouse), PSNPs plus KI (PSNPs + KI) or KI conjugated to PSNPs (PSNPs-KI) (25 µg per mouse KI and 1% solids PSNPs for both groups), or Montanide plus KI (25 µg KI per mouse in 70%/30% *v*/*v* Montanide/PBS). Two weeks after the last immunization, splenocytes from immunized mice were harvested and restimulated with altered peptides in an in vitro ELISpot assay for IFN-γ. Data shown as mean +/- SD of spot forming units (SFU)/million cells per assay triplicates (pooled cells from 3–4 mice per group). * *p* < 0.05, ** *p* < 0.01, *** *p* < 0.001, **** *p* < 0.0001.

Following two immunizations, KI alone and KI plus PSNPs again only produced minimal responses, however immunizing twice with Montanide plus KI induced a similar pattern of response across the peptides, with the highest amount of IFN-γ produced to KI and significant levels of response above media to all altered peptides, apart from A8 (Figure 2). Again, there was cross-reactive activation of T cell responses to all altered peptides, apart from A8, though the responses were significantly lower than to the native KI epitope (Figure 2). Consistent with previous reports, following two immunizations with conjugated KI to PSNPs (PSNPs-KI), the magnitude of the CD8+ T cell response to KI was substantially higher compared to one immunization (Figure 2). Restimulated in vitro cross-reactive IFN-γ responses in the PSNPs-KI group to A8, A5, A4, A3, and A1 were also significantly reduced compared to KI, though all but A8 were significantly higher than background responses (Figure 2). This suggests that most altered epitopes are still able to be recognized by KI primed T cells, though induce lower reactivity. Interestingly, there was no loss of reactivity to the restimulated A7 peptide in the PSNPs-KI group, but not the Montanide plus KI group, following two immunizations. This epitope displayed in vitro cross-reactive IFN-γ responses of a comparable magnitude to the index KI epitope itself (Figure 2). This suggests that conjugation to the nanoparticles is preserving the cross-reactive response to specific altered epitopes, that is not seen with other adjuvants.

### 2.2. Ex Vivo Peptide Pulsed DCs Induce Potent T Cell Responses and Moderate Restimulated Cross-Reactive Responses to Alanine Screened Peptide Variants

**Figure 3:**
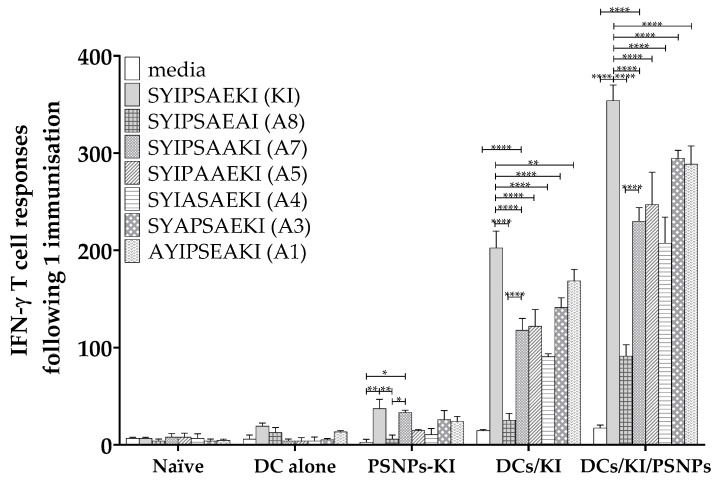
Differential response to alanine screened altered variants of SYIPSAEKI when conjugated to PSNPs or pulsed with DCs. The 6–8 week old BALB/c mice were immunized once with PBS alone, DCs alone (1 × 10^6^ DCs per mouse), KI conjugated to PSNPs (PSNPs-KI, 25 µg per mouse KI and 1% solids PSNPs), ex vivo KI pulsed DCs (DCs/KI, DCs pulsed with 5 µg/mL KI and 1 × 10^6^ cells immunized per mouse), or ex vivo KI pulsed DCs coinjected with mixed in PSNPs (DCs/KI/PSNPs, 1 × 10^6^ cells injected with 0.67% PSNPs). Five weeks after the last immunization, splenocytes from immunized mice were harvested and restimulated with alanine altered peptides in an in vitro ELISpot assay for IFN-γ. Data shown as mean +/- SD of SFU/million cells per assay triplicates (pooled cells from four mice per group). * *p* < 0.05, ** *p* < 0.01, *** *p* < 0.001, **** *p* < 0.0001.

It is possible that the cross-reactivity observed above might be partially due to the peptide conjugations to the PSNPs which could alter the amino acid recognition or enhance/decrease amino acid accessibility during MHC presentation to the TCR. To further explore the mechanism of this cross-reactive response, peptide antigen delivery by DCs was examined. Mice were immunized once with another strong T cell inducing delivery system, autologous ex vivo dendritic cells pulsed with the KI peptide, then reinjected into mice. Additionally, to test the theory that chemical conjugation of the KI peptide to PSNPs is enhancing the response, peptide pulsed DCs were co-injected with mixed in PSNPs rather than chemically conjugating the peptide to the PSNPs. Though the T cell responses, measured by IFN-γ ELISpot assay, were lower after only a single immunization, PSNPs-KI again induced significant responses to the native KI epitope itself and cross-reactive restimulated responses to A7 (Figure 3). For the ex vivo KI pulsed DC immunized group, there was a strong magnitude response to both the index KI peptide and restimulated cross-reactive responses to all altered variants bar A8 (Figure 3). Co-injecting KI peptide pulsed DCs with mixed in PSNPs elicited higher magnitude recall T cell responses compared to the ex vivo peptide pulsed DCs alone, with responses to all peptides significant above background (Figure 3). Consistently, there was a loss of restimulated IFN-γ reactivity to the altered peptide variants compared to KI restimulated responses in both the ex vivo KI pulsed DC group and the combined KI pulsed DCs mixed with PSNPs groups. This further supports chemical conjugation of the peptides to PSNPs as being able to influence the response to peptide variants with amino acid changes outside the key TCR reactive residue.

### 2.3. PSNPs Conjugated Peptide Variants (KI or A7) Elicit Peptide Specific IFN-γ T Cell Responses and Bi-Directional Cross-Reactivity

**Figure 4:**
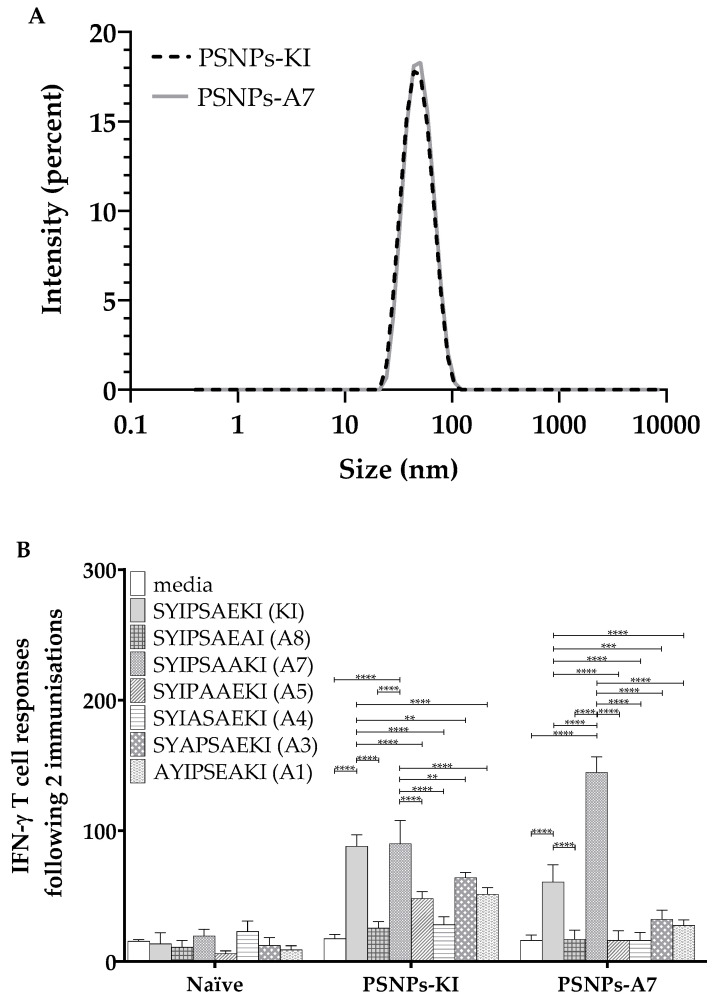
Cross-reactivity to both SYIPSAAKI (A7) and SYIPSAEKI (KI) when either peptide is conjugated to PSNPs. KI and A7 peptides were conjugated to PSNPs and sizes measured on a Malvern zetasizer (**A**). The 6–8 week old BALB/c mice were immunized twice with PBS alone or KI or A7 conjugated to PSNPs (25 µg per mouse KI and 0.72–0.92% solids PSNPs for both groups) (**B**). Thirteen days after the last immunization, splenocytes from immunized mice were harvested and restimulated with altered peptides in an in vitro ELISpot assay for IFN-γ. Data shown as mean +/- SD of SFU/million cells per assay triplicates (pooled cells from four mice per group). * *p* < 0.05, ** *p* < 0.01, *** *p* < 0.001, **** *p* < 0.0001.

To further assess the reactivity of the conjugated peptide responses that was not influenced by alteration of position A7, this peptide was chemically conjugated to the PSNPs and compared to KI conjugated PSNPs immunized responses. The z-average (d.nm) size of both chemically conjugated peptides following dialysis in PBS were 46.79 nm (PdI: 0.075) for PSNPs-KI and 45.51 nm (PdI: 0.090) for PSNPs-A7 (Figure 4A), which showed highly uniform sized and unaggregated PSNPs-peptide formulations. Peptide conjugation efficiency for both peptides were similar (see Table 2 in Section 4). As above, mice immunized with PSNPs-KI elicited a high magnitude IFN-γ T cell response to both KI and A7, as well as modest restimulated cross-reactive responses to A5, A3, and A1, with these altered variant restimulated responses significantly lower in comparison to KI (Figure 4B). In comparison, mice immunized with A7 conjugated to PSNPs elicited a strong IFN-γ response to the A7 peptide itself, as well as a moderate cross-reactive response to the index epitope KI (Figure 4B). Immunizing with PSNPs conjugated to A7 induced bi-directional cross-reactivity to KI and A7, with all other restimulated responses to the altered peptide variants abrogated in the PSNPs-A7 immunized group.

### 2.4. In Vitro Restimulation of Immunized Splenocytes with SYIPSAEKI co-Incubated with the Alanine Variants Together Shows no Evidence of Antagonistic Responses

**Figure 5:**
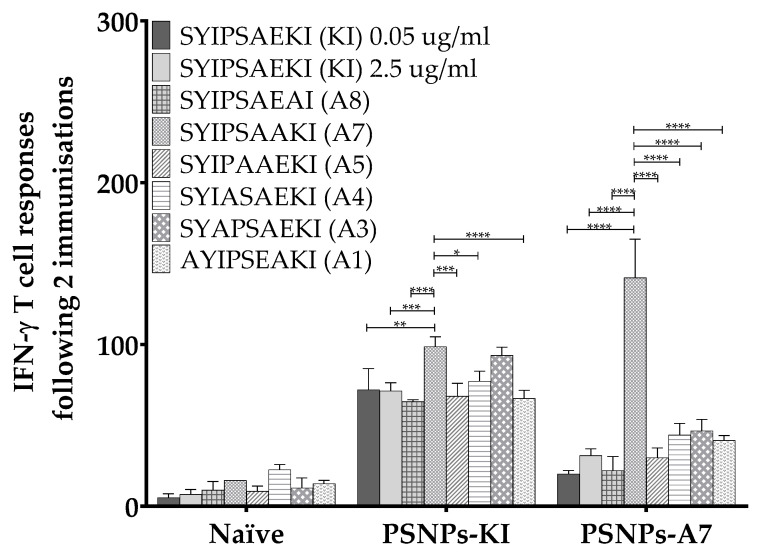
No evidence of antagonistic responses for KI restimulated together with alanine altered peptide variants in vitro. The 6–8 week old BALB/c mice were immunized twice with PBS alone or KI or A7 conjugated to PSNPs (25 µg per mouse KI and 0.72–0.92% solids PSNPs for both groups). Thirteen days after the last immunization, splenocytes from immunized mice were harvested and restimulated with KI for one hour followed by addition of each alanine altered peptide for another ~16 hours in an in vitro ELISpot assay for IFN-γ. Data shown as mean +/− SD of SFU/million cells per assay triplicates (pooled cells from four mice per group). * *p* < 0.05, ** *p* < 0.01, *** *p* < 0.001, **** *p* < 0.0001.

Due to the observed bi-directional cross-reactivity of both KI to A7 and vice versa, it was next examined if there was any evidence of peptide antagonism induced by restimulating the peptides together. To investigate this, mice were immunized with PSNPs conjugated to either KI or A7 as above, and the cross-reactive peptide responses were restimulated in vitro in the ELISpot assay to a suboptimal dose of KI first, before being co-incubated with each of the other peptide variants. Moderate responses were seen from mice immunized with PSNPs-KI to the suboptimal dose of KI alone, with no antagonistic responses observed to any of the altered peptides (as would be indicated by a decreased response) (Figure 5). Additionally, for the PSNPs-KI immunized group, coincubation with both KI and A7 slightly enhanced the restimulated response to A7, significantly higher than all other peptides, apart from A5 (Figure 5). Cross-reactive restimulated responses from mice immunized with PSNPs-A7 were lower for the suboptimal dose of KI, and no antagonism was observed for any of the altered peptides. In fact, for PSNPs-A7 immunized mice, in vitro coincubation of KI and A7 induced a significantly higher level of IFN-γ response to A7 compared to KI alone, higher than all other peptide responses (Figure 5).

## 3. Discussion

Previously it has been shown that minimal immunodominant peptide epitopes from *Plasmodium berghei* (SYIPSAEKI; abbreviated to KI) could induce high magnitude CD8+ T cell responses when used with potent adjuvant systems [23], and it is known that the lysine (K) in position 8 is a key T cell recognition site [24]. This study aimed to identify whether altering amino acid residues outside known T cell reactive sites along the peptide would influence the T cell response, through the process of alanine screening. Here, what has been shown is that by altering residues along minimal peptide epitopes other than the T cell recognition site can influence the immune response and bi-directional cross reactivities. Amino acids in the peptide KI at the position 7, glutamic acid (E); position 5, serine (S); position 3, isoleucine (I) and position 1 serine (S) along the epitope may be involved in the T cell recognition motif as changing each of these residues to an alanine decreased T cell re-stimulation in vitro. 

Furthermore, the choice of adjuvant or delivery system can also modify the immunogenic response. Aside from position 8, the re-stimulated response to all other alanine screened peptide variants was modest in the three adjuvant/delivery systems tested, suggesting that the only residue that fully abrogates a cross-reactive response when altered is at position 8, the known T cell recognition and binding site [24,25]. However, conjugating peptide KI to the PSNPs produced an equivalent cross-reactive response to peptide A7, following two immunizations, comparable to the response to the peptide KI itself. Additionally, it was observed that chemically conjugating A7 to the PSNPs results in bi-directional cross-reactivity between KI and A7. As a model, this would imply that designing vaccines using identified altered variants, or even rationally designed epitopes, may be useful to boost responses to a specific variant of interest, whilst also inducing responses to the index immunodominant epitope. The disadvantage thus far is that this may not result in a broadening of the cross-reactive response to multiple variants in a population. 

The observed cross-reactive response to the native epitope is promising, though the phenomenon of altered peptide ligand antagonism when two peptide variants are presented together was also a possibility, and has been documented previously to minimal malaria peptide epitopes [21,22]. Antagonistic responses, or peptide interference, occurs when primed responses to one epitope may be diminished by subsequent exposure to APLs [19]. Fortunately, antagonistic responses were not observed in this model to any of the altered epitopes when presented together in vitro. Indeed, the opposite was shown where there was evidence of a synergistic restimulated response between the index KI peptide and A7, elicited from A7 peptide (conjugated to PSNPs) primed cells. 

Taken together, these results suggest altered peptides outside the known key TCR contact site should be considered as important parts of the motif for influencing the immunogenic response. Additionally, PSNPs conjugated to immunodominant peptide epitopes or APLs are a potent delivery system that induce high magnitude T cell responses comparable to other adjuvant systems, following two immunizations. Whilst Montanide emulsion and peptide pulsed DCs are potent delivery systems following a single immunization, peptide conjugated PSNPs require a boost immunization to induce such high magnitude responses. This is consistent with previous reports of peptide conjugated nanoparticles [23]. Though PSNPs require two immunizations for high magnitude responses, they offer protective immunity to a range of pathogens [16,27,28] and do not induce classical inflammatory cytokines or suppressive cell subsets, such as myeloid derived suppressor cells (MDSCs) and tumor necrosis factor receptor 2 (TNFR2)+ T regulatory cells, compared to other delivery systems, including Montanide [15,23]. This study has also shown peptide conjugation to this nanoparticle delivery system preserves specific APL responses.

### 3.1. Influence of Conjugation on Peptide Responses

There are multiple possibilities as to why there is a difference in the immune response to peptide “conjugated” to or “mixed” with the PSNPs. There may be differences in the way the peptides conjugate to the PSNPs themselves. The peptide is expected to conjugate at the N terminus to form an amide with the activated carboxyl groups on the nanoparticles. Due to both the peptides and nanoparticles being exposed to the EDC chemical reaction, there is the possibility of side reactions and the potential formation of peptide multimers attached to the PSNPs. The activated carboxyl group on the glutamic acid in position 7 in the KI epitope and the C-terminal carboxyl group of both KI and A7 are all possible sites of conjugation. Therefore, there are two possible amino groups that can conjugate to the particles for KI and one amino group for A7. It is expected that this could influence the strength of the induced response as it may lead to secondary structure formation, or multilayer peptide conjugations. Whilst multimer formation is possible, the small size (45–46 nm) and monodisperse nature of the peptide-nanoparticle conjugates, compared to naked nanoparticles (≈43–44 nm [15]) suggest that larger aggregated peptide complexes are not forming. 

Conjugating the peptide at the C terminus with the T cell recognition site further away from the particle may also be a factor in the resulting immune response. Though, in a previous study longer peptides were compared, both CD8 and CD4 epitopes of OVA linked together, and showed that there was no significant difference in the response if the CD8 epitope was closer or further away from the nanoparticle [27]. Interestingly, this study used the model SIINFEKL epitope, which also has a lysine at the C-terminal end, adjacent to the MHC anchor residue. For SIINFEKL it has also been determined that altering residues within the binding groove (i.e., position 2), can affect TCR recognition, without influencing binding, suggesting conformational changes [29].

### 3.2. Differential Uptake of Conjugated vs. Soluble Peptides

Cellular uptake of antigenic peptides from the vaccination site is performed by specialized antigen-presenting cells (APCs) with efficient proteolytic processing for MHC I and class II presentation to CD8+ T cells or CD4+ T cells, respectively. Peptides are typically difficult to internalize into cells due to their hydrophilicity. Therefore, for entry into cells, peptides are better off linked to a carrier. Peptides mixed with PSNPs or conjugated to PSNPs will have differential uptake efficiency and endosomal antigen localization by APCs. Covalently conjugated peptides to PSNPs will be more stable and taken up by cells, whereas peptide mixed PSNPs are taken up independently or only joined by physical adsorption. Depending on the surface charges of the PSNPs and the net charge of peptide, the binding is usually neither strong nor stable. The KI peptide is highly hydrophilic, with the PI of 6.58 (Table 1, see Section 4). Therefore, in a physiological condition (pH ≈ 7.4), the net charge of the KI peptide is ≈0, which would be theoretically difficult to adsorb to the carboxylated PSNPs used in this study based on the charges. Hence, the conjugated peptide KI to PSNPs may be internalized by APCs much more efficiently than the same amount of free KI peptides. The peptides conjugated to PSNPs are usually endocytosed and canalize intracellular trafficking toward early endosomal low-degradative compartments rather than lysosomes for degradation [30]. This favors antigen cross-presentation and consequently enhances CD8+ T cell activation [30]. Therefore, conjugation of peptide KI to PSNPs would increase DC uptake as well as antigen processing and presentation on MHC class I molecules.

The sequence and amino acid composition may also alter the physical properties and immunological behavior of individual peptides, which likely alters the way a peptide is internalized, processed, and presented. Peptide A7 has an overall positive charge, and the naked peptide A7 should have better uptake by the APCs than the naked peptide KI without conjugation to PSNPs.

### 3.3. Peptide Recognition by the TCR

Another reason for the difference in response to conjugated epitopes may be the way the peptide structurally binds to the MHC-TCR complex. To date, the structure of KI in the H2-Kd binding groove has not been definitively solved but aspects of the structure have been modelled by others. It has also been compared to other structures of H2-Kd restricted peptide epitopes, such as the influenza nucleoprotein (NP) peptide [31]. Of note in an influenza NP peptide structure model, position 7 along the peptide faces towards the α2 helix, whereas position 8 along the peptide faces in the opposite direction towards the α1 helix [31]. In future, it would be of interest to investigate whether the altered residues at this position, or any of the alanine screened peptides, can bind strongly to the TCR, including if there are any differences in binding of peptides that have undergone chemical conjugation. Additional investigation of immunogenic responses to altered amino acids other than alanine may also provide further insight into the peptide recognition properties.

Previously studies have shown that similar alanine altered variants alone can bind and are recognized by the TCR at altered positions A3, A4, A5, and A7 [25]. One study fingerprinting HLA-A2.1 TCR peptides showed that positions 1 and 3 were not affected by amino acid variations and positions 5 and 8 were crucial for TCR recognition as they face the TCR [32]. They also identified positions 4, 6, and 7 to have a role in TCR recognition [32]. This is consistent with what has been observed for KI peptides regarding position 8 abrogating the response and altering positions 1, 3, 5, and 7 permitting T cell reactivity, though at lowered levels of response. Conversely, others have indicated that positions 4, 5, and 6 are crucial TCR recognition sites, as the largest difference in response was seen when these positions were altered [33].

### 3.4. Influence of Each Amino Acid Position along the Peptide

Others have predicted that specific characteristics of each amino acid, for example if they are hydrophobic [34] or have a particular shape such as an aromatic ring, determines TCR recognition. Understanding this information is important for the basis of epitope prediction programs. This does not seem to be the case with the KI epitope as the lysine in position 8 and glutamic acid in position 7 are not hydrophobic. Furthermore, when these residues were changed to a hydrophobic alanine at either A8 or A7, TCR recognition was lowered or diminished (apart from when A7 was bound to PSNPs). One other main theory is that changing an amino acid adjacent to the main TCR contact site affects the shape of the epitope, altering the response. Previous studies have discussed the interactions of the peptides, i.e., that positions 4 and 5 potentially interact with each other plus the MHC, resulting in positions 4 and 5 facing away from the MHC towards the TCR [32]. Based on the findings in our study, it is speculated that the interactions of KI conjugated to the nanoparticles, or possible A7 and K amino acid interactions in the altered epitope, account for the differences in the observed cross-reactive response to A7. Whilst this is not apparent when KI is mixed with soluble adjuvants, chemical conjugation of KI may change the shape or side charge contacts of position 7, potentially making it more assessible for recognition or easily activated by the TCR for cross-reactivity. Investigating the kinetics of TCR recognition and activation (indicated by immunogenicity responses), is important for design of peptides for potent subunit vaccines against complex pathogens.

Understanding the effects of each amino acid residue and the influence of peptide delivery systems, i.e., by chemical conjugation to nanoparticles, would give an added advantage of designing novel altered peptide ligand bound nanovaccines for pathogens that readily mutate and may need super agonist epitopes to induce potent T cell responses. The current model uses the example of liver stage malaria epitopes, though these findings would also be applicable to other diseases, such as viral infections or cancer, where a strong CD8+ T cell response correlates with favorable outcomes.

## 4. Materials and Methods

### 4.1. Animals

Six to eight week old BALB/c mice from Monash Animal Services were used under ethical approval (14 May 2014, ethics number E/1454/2014/M) by the Alfred Medical Research and Education Precinct (AMREP) Animal Ethics Committee (AEC).

### 4.2. Peptides

Alanine screened peptides SYIPSAEKI, SYIPSAEAI, SYIPSAAKI, SYIPSAEKI, SYIASAEKI, SYAPSAEKI, AYIPSAEKI (Table 1) were synthesized by Mimotopes (>95% purity, H- and –OH termini for the N- and C-terminals, respectively, Mimotopes, Melbourne, Australia).

### 4.3. Peptide Conjugation to PSNPs

SYIPSAEKI and SYIPSAAKI were conjugated to carboxylated PSNPs (Polysciences Inc., Warrington, PA, USA) based on the method previously described [23,24]. PSNPs (1% solids) were activated using 1-ethyl-3-(3-dimethylaminopropryl) carbodiimide hydrochloride (EDC, 4 mg/mL) in MES buffer (50 mM, pH 7), together with either peptide (1 mg/mL), and mixed on a rotary wheel for 4 h at room temperature. The carboxyl sites at the C-terminal on each peptide and the glutamic acid side chain in position 7 of SYIPSAEKI may also be activated in this reaction. The reaction was stopped by the addition of glycine (7 mg/mL final, Sigma-Aldrich, St. Louis, MO, USA) and incubated for a further 30 min on the rotary wheel. The conjugation mix was dialyzed in 10–14 kDa cutoff membrane (Viskase, Lombard, IL, USA) against PBS overnight at 4 °C. Conjugation efficiency was determined by BCA assay (Pierce, Thermo Fisher, Waltham, MA, USA) following manufacturer’s instructions and size determined by dynamic light scattering (Zetasizer, Malvern Instruments, Malvern, UK) (Table 2).

### 4.4. Ex vivo DC Peptide Pulsing

Dendritic cells (DCs) were cultured and pulsed with peptide as previously described [24]. Haemopoietic stem cells were harvested from the bone marrow of the femur and tibia of BALB/c mice and seeded at 0.5 × 10^6^ cells /mL in RPMI (Gibco, Thermo Fisher, Waltham, MA, USA) supplemented with 10% FBS, 100 units/mL penicillin, 100 µg streptomycin, 2 mM L-Glutamine, 1 M Hepes (all Gibco, Thermo Fisher), and 0.1 mM 2 mercaptoethanol (Sigma Aldrich, St. Louis, MO, USA), plus 10 ng/mL GM-CSF (PeproTech, Cranbury, NJ, USA) and 5 ng/mL IL-4 (PeproTech, Cranbury, NJ, USA) for 6 days with half the media replaced on day 3. Cultured DCs were pulsed with 5 µg/mL SYIPSAEKI for 1 h and then washed with PBS and resuspended at 1 × 10^7^ cells/mL in PBS.

### 4.5. Vaccines and Immunizations

Vaccines consisted of SYIPSAEKI (final 5 µg /mL) pulsed DCs (1 × 10^6^ cells/mouse in 100 µL PBS) with or without the addition of PSNPs (additional 50 µL at 2% solids), SYIPSAEKI (final 25 µg/mouse) mixed with the adjuvants Montanide ISA 720 (70% *v*/*v* with PBS; supplied by Tall Bennett Group, Sydney, Australia). Nanovaccines contained either SYIPSAEKI (25 µg/mouse) mixed with PSNPs (1% final solids), or peptides covalently conjugated to the PSNPs (25 µg peptide/mouse). Mice were immunized with respective formulations, either once, or twice, two weeks apart, intradermally at the base of the tail. Approximately two weeks (unless otherwise stated) after the last immunization mice were humanely euthanized and splenocytes harvested and assessed for IFN-γ production by ELISpot assay.

### 4.6. ELISpot Assay

T cell responses were assessed by IFN-γ ELISpot assay as described previously [10,11]. Ninety-six well multiscreen plates (MSIP, Merck Millipore, Burlington, MA, USA) were coated with 5 ug/mL anti-mouse IFN-γ (AN18, MABTech, Nacka Strand, Sweden), 100 uL/well in PBS, and incubated overnight at 4 °C. Wells were washed five times with PBS and blocked with complete media for at least 1 h at 37 °C. Splenocytes processed from immunized animals were plated in triplicate wells at 1 × 10^7^ cells/mL (50 µL/well) and incubated with the recall peptides SYIPSAEKI, SYIPSAEAI, SYIPSAAKI, SYIASAEKI, SYAPSAEKI, and AYIPSAEKI (final 2.5 µg/mL). Media alone and concanavalin A were used as negative and positive controls, respectively. Where indicated, cells were first co-incubated with SYIPSAEKI at 0.05 µg/mL for 1 h, before the addition of the altered peptides in respective wells (according to plate setup) at a final of 2.5 µg/mL. Plates were incubated at 37 °C, 6% CO_2_ for ≈ 16 h. Plates were then washed with PBS and biotinylated anti-IFN-γ (R4-6A2-biotin, MABTech) added to each well (100 µL in PBS/0.5% FBS) for 2 h at RT. Plates were washed five times in PBS and streptavidin-alkaline-phosphate enzyme conjugate (Strep-ALP, MABTech, 1 µg/mL in PBS/0.5% FBS, 100 µL/well) added for 1.5 h at RT. Plates were given a final wash with PBS and reverse osmosis water before spots were developed for 30 min using an AP colorimetric kit (Bio-Rad, Hercules, CA, USA), following the manufacturer’s instructions. Plates were imaged and counted on an AID ELISpot reader system (AutoImmune Diagnostika GmbH, Straßberg, Germany).

### 4.7. Statistics

All statistics were performed using GraphPad Prism (v.6). Two-way analysis of variance with post hoc Tukey multiple comparison analysis were performed, with statistical significance set at *p* < 0.05. Values are expressed as mean +/− standard deviation (SD). Group sizes and replicates are indicated in the figure legends.

## 5. Conclusions

Whilst more is known about the vaccine response to peptides with changes in key T cell contact residues, it has now been shown that altering amino acids outside of known T cell recognition residues and MHC anchor residues can influence the vaccine induced T cell response. Furthermore, altered epitopes may show an advantage in enhancing a specific cross-reactive response when used with particular delivery systems. Subunit peptide vaccines are advantageous as they are cost effective and practical to synthesize. Identifying or designing highly immunogenic epitopes that are recognized by the TCR and induce potent immunogenicity would benefit from understanding which positions along the epitope contribute to the response and whether or not alterations in these residues interfere with immunogenicity.

## Figures and Tables

**Table 1:** Alanine substitutions along the SYIPSAEKI peptide epitope.

Designated Name	Peptide Sequence ^1^	PI^2^	Net Charge at pH 7
KI	SYIPSAEKI	6.58	0
A8	SYIPSAEAI	0.94	−1
A7	SYIPSAAKI	9.5	1
A5	SYIPAAEKI	6.58	0
A4	SYIASAEKI	6.58	0
A3	SYAPSAEKI	6.58	0
A1	AYIPSAEKI	6.84	0

^1^ Bold amino acid highlights the change to an alanine. ^2^ PI is the isoelectric point.

**Table 2:** Characterization of peptides conjugated to the PSNPs.

	Peptide Purity ^1^	Concentration of Conjugated Peptide ^2^	# of Peptide Molecules per Particle ^3^	Size of PSNPs after Conjugation ^4^	PdI ^5^	% PSNPs Solids Injected ^6^	µg Peptide Injected per Mouse
**PSNPs-KI**	>95%	0.21 mg/mL	1047.77	45.29 ± 1.3 nm	0.077	0.92%	25 ug
**PSNPs-A7**	>95%	0.29 mg/mL	1390.92	46.59 ± 1.4 nm	0.075	0.72%	25 ug

^1^ The peptide purity was defined when ordered. ^2^ The concentration of conjugated peptide was determined by BCA assay. ^3^ The number of peptides per particle was calculated by working out the number of molecules per mL (number of antigen molecules per mg multiplied by the concentration of conjugated peptide in mg/mL) relative to the number of particles per mL. ^4^ The size of the particle conjugates were measured on a Malvern Zetasizer, z-average size is reported. ^5^ The PdI is the polydispersity index measured on the zetasizer (a small PdI indicates a narrow size distribution). ^6^ The percentage of particles solids injected was calculated by the volume required to inject 25 µg peptide per mouse, following conjugation and dialysis.

## Abbreviations

A1	AYIPSAEKI peptide
A3	SYAPSAEKI peptide
A4	SYIASAEKI peptide
A5	SYIPAAEKI peptide
A7	SYIPSAAKI peptide
A8	SYIPSAEAI peptide
AEC	Animal Ethics Committee
AMREP	Alfred Medical Research and Education Precinct
APCs	Antigen presenting cells
CD	Cluster of differentiation
ChAd	Chimpanzee adenoviral
CO_2_	Carbon dioxide
DCs	Dendritic cells
EDC	1-Ethyl-3-(3-dimethylaminopropyl)carbodiimide
FBS	Fetal bovine serum
GM-CSF	Granulocyte-macrophage colony-stimulating factor
IFN-y	Interferon gamma
IL	Interleukin
ISCOMs	Immune stimulating complexes
kDa	Kilodalton
KI	SYIPSAEKI peptide
MDSCs	Myeloid derived suppressor cells
MES	2-(N-morpholino) ethanesulfonic acid
MHC	Major histocompatibility complex
MVA	Modified vaccinia Ankara
NP	Nucleoprotein
PBS	Phosphate buffered saline
PSNPs	Polystyrene nanoparticles
RT	Room temperature
SD	Standard deviation
SFU	Spot forming unit
TCR	T cell receptor
TNFR2	Tumour necrosis factor receptor 2
VLP	Virus like particle
